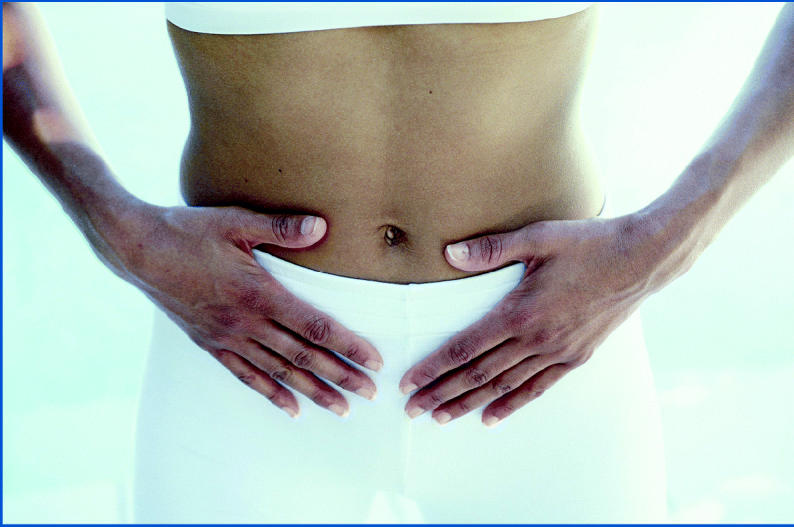# Headliners: Uterine Leiomyoma: Genetic Reprogramming and Benign Uterine Tumors

**Published:** 2005-11

**Authors:** Jerry Phelps

Cook JD, Davis BJ, Cai SL, Barrett JC, Conti CJ, Walker CL. 2005. Interaction between genetic susceptibility and early-life environmental exposure determines tumor-suppressor-gene penetrance. Proc Natl Acad Sci USA 102:8644–8649.

Uterine leiomyomas (fibroids) are common benign tumors in the muscle tissue of the uterus. Previous research has suggested a link between environmental exposures and uterine fibroids. NIEHS grantee Cheryl Lyn Walker and colleagues at The University of Texas M.D. Anderson Cancer Center were interested in how such exposures contribute to uterine fibroids. They propose that early-life exposure to xenoestrogens may alter genetic programming during development, setting the stage for an adverse response to later natural estrogen stimulation.

Uterine fibroids occur in up to 77% of women, can cause severe menstrual bleeding and pelvic discomfort, and result in more than 200,000 hysterectomies each year in the United States alone; although “benign,” they are far from harmless. Lesions causing symptoms range in size from 1 to 20 centimeters. Data indicate that 25% of white women have problematic lesions. Black women have about a threefold higher risk of developing fibroids and, in general, their clinical symptoms are worse.

Diethylstilbestrol (DES), a xenoestrogen, is one environmental exposure that has been posited as contributing to uterine fibroids. To determine the actions of this chemical, Walker and colleagues studied rats with a genetic predisposition to developing uterine fibroids, exposing some of them to DES during their first week of life. By age 16 months, the DES-exposed animals had almost a 95% incidence of tumor formation, while the unexposed animals had a 64% incidence. There were more tumors in each affected DES-exposed animal, and the tumors were larger in size and more invasive, compared to controls.

The researchers determined that DES did not cause a mutation in estrogen-responsive genes, but rather caused them to become “reprogrammed” so that they responded differently to natural estrogen stimulation later in life. These findings indicate that reprogramming of genes during the developmental period as a consequence of xenoestrogenic exposure can interact with a preexisting genetic condition to increase the formation and severity of uterine fibroids. If additional research confirms these results, this study’s findings could have implications for other hormonally mediated cancers such as those of the breast and prostate.

## Figures and Tables

**Figure:**